# The National Institute of Open Schooling

**DOI:** 10.4103/0019-5545.43618

**Published:** 2008

**Authors:** Chittaranjan Andrade

**Affiliations:** Department of Psychopharmacology, National Institute of Mental Health and Neurosciences, Bangalore - 560 029, Karnataka, India

The National open School or the National Institute of Open Schooling (NIOS; www.nos.org) is an important initiative of the Government of India, created to cater to the educational needs of students who cannot attend regular schools. Students who benefit from this initiative include sportspersons who have to train and travel all through the academic year; those with a physical handicap; and those with chronic medical illness. Additionally, a large number of candidates who register with the NIOS Board are those with learning disorders or other psychiatric conditions.

## THE NIOS BOARD

The NIOS has been in existence since 1990. It offers a national board examination at two levels: secondary and higher secondary. The secondary level is equivalent to the ICSE, CBSE, SSLC, and other national or state Standard 10 board examinations. The higher secondary level is equivalent to the PUC and other Standard 12 board examinations. Many (but not all) boards, institutions, and universities across the country recognize the NIOS certification for continued or higher studies; these are listed in the NIOS prospectus and at the NIOS website. However, as those who could not attend regular school may not be able to attend regular college either, the Indira Gandhi National Open University (IGNOU) may be an opportunity for continuing education for students who complete their higher secondary studies with the NIOS.

## NIOS SUBJECTS

Regular schools require students to study all the subjects in the curriculum: two languages, science, mathematics, and social studies; each subject may have more than one paper. The school boards with which regular schools are registered allow, at the most, an exemption from second language for students with a language disability; and some boards require the magnitude of this disability to exceed 50% to qualify for the exemption. No national board and probably no state board allow exemptions from science or mathematics, such as for students with arithmetic disorder.

In contrast, the NIOS requires students to study a minimum of five and a maximum of seven subjects. There is a very wide range of subjects on offer; students, therefore, have much flexibility in selecting areas in which they are able to attempt an examination. Students are also allowed to change subjects midway through the course if they are unhappy with their choice. Students can choose from among three languages for their medium of instruction.

The secondary examination subjects can be chosen from the following list: any one or two of 15 different languages mathematics, science, social studies, economics, business studies, home science, typewriting (either of two languages), word processing (either of two languages), psychology, and Indian culture and heritage.

The senior secondary examination subjects can be chosen from the following list: any one or two of three different languages; mathematics, physics, chemistry, biology, history, geography, political science, economics, commerce, accounts, home science, typewriting (any of three languages), word processing, stenography (any of three languages), secretarial practice, psychology, computer science, sociology, and painting.

## REGISTERING WITH THE NIOS

Students who fulfill the specified minimum age criteria can register for the secondary or higher secondary NIOS courses during July and August in each calendar year; the exact dates are specified at the website. Students can register for the secondary course only if they have successfully completed their education to at least a Standard 8 level in a regular school; however, in its wisdom the NIOS board allows candidates to self-certify their fulfillment of this criterion without requiring it to be proven. Students can register for the higher secondary course only if they have successfully completed their secondary education with a recognized board, including the NIOS secondary board.

More than 2000 institutions in major towns and cities across the country are NIOS-accredited; these institutions are listed in the NIOS prospectus and at the NIOS website. Students can register at these institutions and may receive special tuitions at these institutions or may study from the supplied textbooks in the privacy of their homes; thus, there is no requirement for compulsory attendance. The cost of the registration and of the courseware is low; all sections of society will be able to afford an NIOS education. Instruction is also available from priced audio and video cassettes, compact discs, radio broadcasts, and television broadcasts.

## THE NIOS EXAMINATIONS

Examinations are conducted twice a year: in April-May, and in October-November. Students are allowed a maximum of nine chances across a maximum of five years to complete their examinations; they can appear for as few or as many subjects at a time as they wish. Students who do not complete the examinations in all their chosen subjects within the specified period can re-apply and have their examination credits transferred.

Most questions in the theory papers require short answers, or answers in points. Practical examinations are conducted in subjects such as science, geography, word processing, home science, and painting. The examiners tend to be lenient in their standards, understanding the special needs of the students who appear. Students require to score a minimum of 33% to pass an examination.

The NIOS also offers an on-demand examination system. Thus, a student who completes his preparation in one of his chosen subjects can visit the NIOS center and ask for an immediate examination in that subject. A question paper will be generated from a question bank and he will be examined. The advantages of this system are enormous: the student can appear when he is ready; the pressure on the student is less; and the student is permitted to appear as often as he wants if he wants to improve his performance. At present, though, the on-demand examination system is available only for certain subjects, and only at the NIOS headquarters in Noida. The system is shortly expected to be expanded to included more subjects and other centers across the country.

## THE NIOS WEBSITE

The NIOS website (www.nos.org) is not very well constructed, but adequately serves its purpose. It provides the full text of the prospectus with all the information necessary about the courses. Courseware is available online. Sample question papers and the question papers of previous years are also available online. Important events are periodically announced and updated. The results are announced online.

Whereas there is scope for improvement in the efficiency of the NIOS system, it is overall a scheme of which the country can be proud. Disadvantaged children can at last have an opportunity for pride in having completed their basic education.

